# Green self-assembly of zein-conjugated ZnO/Cd(OH)Cl hierarchical nanocomposites with high cytotoxicity and immune organs targeting

**DOI:** 10.1038/srep24387

**Published:** 2016-04-14

**Authors:** Hua-Jie Wang, Ying Cao, Cai-Feng Wang, Shi-Zhong Cui, Li-Wei Mi, Teruo Miyazawa

**Affiliations:** 1 Center for Advanced Materials Research, Zhongyuan University of Technology, No. 1 Huaihe Road, Xinzheng Shuanghu Economic Development Zone, Zhengzhou 451191, P. R. China; 2 New Industry Creation Hatchery Center (NICHe), Tohoku University, Sendai 980-0845, Japan

## Abstract

Inorganic nanomedicines in the fight against cancer have progressed rapidly during recent years, with the synergistic advantages of multifunctional nanosystems compared to single component. Herein, a drug-combination opinion was introduced into “nanomedicine” based on the understanding of Trojan horse-anti-tumor mechanism of inorganic nano-medicines. Moreover, we reported the green and facile synthesis route of mono-dispersed and rod-like zein-conjugated ZnO/Cd(OH)Cl hierarchical nanocomposites. We found that the nanocomposites exhibited high-efficiency killing ability to tumor cells through lipid peroxidation mediated-membrane disintegration route. The safety studies in *BALB/c* mice didn’t detect injection anaphylaxis, hemolysis and cytotoxicity. More interestingly, the nano-composites could specially accumulate in liver and kidney, which will be helpful for targeting cure to these regional cancers.

Nowadays, inorganic nanomedicines in the fight against cancer are occupying an important place due to the synergistic advantages of multifunctional nanosystems compared to single component^1^,^2^,^3^,^4^,^5^,^6^,^7^,^8^,^9^,^10^. Especially, some nanomaterials can induce physiological function disorder of tumor cells and directly kill them^11^,^12^,^13^,^14^,^15^,^16^,^17^,^18^. For example, Premanathan *et al.* documented that acicular ZnO with 10–30 nm sizes could selectively kill human myeloblastic leukemia HL60 cancerous cells through lipid peroxidation, but spare normal peripheral blood mononuclear cells^19^. Up till now, a high anti-tumor activity of nanomedicines while simultaneously minimizing the side effects to normal tissues is becoming a major challenge of tumor therapy. It drives the revolution of anti-tumor drugs.

Of the various therapeutic strategies available for tumors, drug combination is one of the most effective ones in which more than one medicine are administered to patient, and may produce effects that are greater than or less than the effect predicted from their individual potency^20^. The aims of the present work were to introduce the drug-combination opinion into “nanomedicine” and to design a green pathway for synthesis of zein-conjugated ZnO/Cd(OH)Cl hierarchical nano-composites (Z-ZnO/Cd(OH)Cl nano-composites). Here, the green synthesis pathway consisted of the biomimetic synthesis and ion exchange process as shown in Fig. 1 and would be realized under the aqueous system and gentle conditions. Like *in vivo* reaction, biomimetic synthesis uses native or synthetic molecules as templates to control the hierarchical assembly of nano-materials with special shape, size and pholymorph under ambient condition in aqueous environments^21^,^22^,^23^,^24^,^25^,^26^,^27^. In this work, we selected zein as the structure-directing agent to control the formation of nanocomposites for the following reasons: (1) zein is a predominant storage protein of corn, which will supply abundant and low-cost raw material^28^. (2) zein is famous in the medicine field due to its excellent biocompatibility^29^,^30^,^31^,^32^,^33^,^34^. (3) The structure of zein has been well documented and it will be helpful to understand the interaction of zein and nanomaterials^35^,^36^. Additionally, based on the understanding of Trojan horse-anti-tumor mechanism of inorganic nano-medicines, after entry of the nanocomposites into tumor cells, zein will be biodegraded. Meanwhile, ZnO and Cd(OH)Cl will be released^33^,^34^,^37^. Then ZnO and Cd(OH)Cl can play a role of drug combination because of the potential and different cytotoxic activity of Zn and Cd or their corresponding compounds to tumor cells^38^,^39^,^40^,^41^. That is to say, the resulting nanocomposites may act as a new anti-tumor agent. The synthesis route was clearly monitored and the anti-tumor activity was confirmed *in vitro*. Besides, a reasonable explanation to the anti-tumor activity was also investigated. The pharmacokinetics and bio-distribution of Z-ZnO/Cd(OH)Cl nanocomposites were evaluated *in vivo*. All the results supported the conclusion that the application of drug-combination opinion in nanomedicine was feasible to find a new anti-tumor drug. Moreover, zein-conjugated ZnO/Cd(OH)Cl hierarchical nanocomposites had the potential as targeting anti-tumor drug.

## Experimental Section

### Biomimetic synthesis of zein-conjugated ZnO nanospindles precursor

Zein-conjugated ZnO nanospindles were prepared by a modified biomimetic synthesis method using zein as the structure-directing agent. Briefly, 15 mL of 20 mg/mL zinc nitrate alcohol aqueous solution (60%) was dropwise added into 40 mL of 1.25 mg/mL zein alcohol aqueous solution (60%) under stirring. The pH value of the mixture was adjusted to 7.5 using 2 M NaOH solution and the mixture was kept static for 3 days at 37 °C. Finally, the as-prepared sample was separated by centrifugation at 10,000 rpm for 10 min. The collected product was washed with double distilled water and vacuum-lyophilized for 8 h.

### Ion exchange for the controllable synthesis of Z-ZnO/Cd(OH)Cl nanocomposites

Z-ZnO/Cd(OH)Cl nanocomposites were fabricated by the ion-exchange method using zein-conjugated ZnO nanospindles as the precursor. Briefly, 12.5 mg zein-conjugated ZnO nanospindles were dispersed in 25 mL of 36.8 mg/mL CdCl_2_ alcohol aqueous solution (60%) and the reaction system was kept at 37 °C for the given intervals. The resulting products were separated by centrifugation at 10,000 rpm for 10 min, rinsed three times with distilled water and vacuum-lyophilized for 8 h.

### Characterization

High-resolution transmission electron microscopy (HR-TEM) observations were carried out in a JEM-2010 electron microscope working at 200 kV for measuring the morphology and microstructure of nanocomposites.

Fourier transform infrared spectra (FTIR) were recorded on a Bio-Rad FTS-40 Fourier transform infrared spectrophotometer in the wavenumber range of 4000-650 cm^−1^. The spectra were collected at 2 cm-1 resolution with 128 scans by preparing KBr pellets with a 3:100 “sample-to-KBr” ratio.

Thermogravimetry-differential scanning calorimetry (TG/DSC) measurement was performed on an EXSTAR TG/DTA 6300 instrument (Seiko, Japan) to quantify protein content in nanocomposites.

The real time analysis was performed according to the crystalline phase and Zn/Cd ratio of nanocomposites by powder X-ray diffraction (XRD, D & Advance, Bruker, Germany) and atomic absorption spectroscopy (AAS, Z-5000, Hitachi, Japan). To determine the crystalline phase, XRD measurements were performed on a Bruker D & Advance X-ray powder diffractometer with graphite monochromatized Cu/Kα (γ = 0.15406 nm). A scanning rate of 0.05 deg/s was applied to record the pattern in the 2θ range of 10–80°. At the same time, the samples were also digested by nitric acid and the Zn and Cd concentrations were quantified by AAS test.

### Exposure of PC12 cells to nanocomposites

PC 12 cells were routinely cultured in tissue culture flasks with high-glucose Dulbeccos’ Modified Eagle Medium (HG-DMEM), containing 10% fetal bovine serum and incubated at 37 °C in a humidified atmosphere with 95% air and 5% CO_2_. The culture medium was refreshed every two days. When the cells became almost confluent, they were released by treatment with 0.25% trypsin. Then the cells were counted to 10^4^ cells/cm^2^ and 200 μL of the cells suspension was pipetted into 96-well tissue culture plate. After 12 h of pre-culture, the medium was replaced with the fresh HG-DMEM medium containing 5–100 ppm of different nanomaterials. In addition, the normal group without any additive was chosen as the control group.

### Cell viability analysis

The cell viability after exposure to different nanocomposites for 48 h was analyzed on the basis of the mitochondrial viability and neutral red uptake assay. The mitochondrial viability was measured by a 3-(4,5-dimethylthiazol-2-yl)-2,5- diphenyl tetrazolium bromide assay (MTT) based on succinic dehydrogenase activity at OD 490 nm (n = 3); 630 nm was chosen as the reference wavelength. Briefly, after 48 h of cell culture, the wells were carefully washed with phosphate buffered saline (PBS, pH 7.2, 0.1 mol/L). 200 μL of 10% MTT in culture medium was added into each well and cell culture was continued for another 4 h. Then the solution was removed and the wells were washed twice with PBS. 200 μL of dimethyl sulfoxide was pipetted into each well and OD values were read on a microplate reader (Multiskan MK3, Thermo Labsystems, USA).

The neutral red uptake assay was used to determine the accumulation of neutral red dye in the lysosomes of the viable. Briefly, the medium was replaced with serum free medium containing 50 μg/mL neutral red dye. After incubation for another 3 h at 37 °C, the wells were washed twice with PBS and then dissolved in a mixing solution of 50% ethanol and 1% acetic acid in Milli-Q water. OD values at 540 nm were read on a microplate reader (Multiskan MK3, Thermo Labsystems, USA).

### Cell membrane damage

The cell membrane damage after exposure to different nanocomposites for 48 h was detected by lactate dehydrogenase (LDH) activity, and microscope observation. Briefly, the LDH in extracellular medium was measured using an LDH assay kit (sigma) according to manufacturer’s protocol. Absorbance was detected at 490 nm in a microplate reader (n = 6) (Multiskan MK3, Thermo Labsystems, USA).

The nucleic acids distribution was observed on the fluorescence microscope (Axioskop 40, ZEISS, Germany) after being stained with acridine orange for 1 min.

As for SEM analysis, the samples were taken out randomly after 48 h of cell culture and washed with PBS (pH 7.2, 0.1 mol/L). Cells were then fixed by immersing the samples into 2.5% glutaraldehyde solution and allowing them to stand in the fixative at 4 °C for over 2 h. Then a standard dehydration in ethanol graded series was performed. Finally samples were mounted on stubs, coated in vacuum with gold and exampled by SEM (JEOL JSM-6390, Seiko, Japan).

As for TEM analysis, the cells were fixed with 2.5% glutharaldehyde and postfixed in 1% osmium tetroxide. After being dehydrated in graded series of absolute alcohol, the cells were embedded in Spurr’s resin (Spurr’s Kit, Electron Microscopy Sciences, Co, USA). Ultra thin sections (50 nm) were made and stained with 1% of uranyl acetate and 0.5% lead citrate. The samples were examined by TEM (FEI-Philips Tecnai G2, Philips, Holland).

### Oxidative stress markers

The level of lipid peroxidation was expressed as malondialdehyde (MDA) content and determined by trichloracetic acid (TCA) reactive substances formation. Briefly, PC 12 cells were exposed to 5 ppm of different nano-composites for 48 h. 40 μL of the cell culture medium was taken out and mixed with 160 μL of the TCA-thiobarbituric acid (TBA) solution (containing 20% of TCA and 0.5% of TBA). The mixture was heated at 100 °C for 20 min and then quickly cooled in ice. After centrifugation at 7, 000 rpm for 10 min, the absorbance of the supernatant was measured at 532 nm (n = 6). The standard curve was prepared by using known concentrations of MDA ranging from 10 nM to 100 μM. By comparison with the control group, the lipid peroxidation was expressed in the relative content of MDA per cell and the value for the control was set as 1.

As for glutathione (GSH) estimation, cells were collected by centrifugation at 2,000 rpm for 10 min at 4 °C. The cell pellet was lysed in cold 20 mM HEPES buffer (pH7.2). Then the solution was centrifuged at 10,000 rpm for 10 min at 4 °C. The protein in the supernatant was precipitated with 1% perchloric acid. After centrifugation treatment at 10, 000 rpm for 5 min, 20 μL the supernatant was mixed with 160 μL of 100 mM phosphate-5 mM EDTA buffer and 20 μL o-phthalaldehyde (1 mg/mL in methanol). The reactive system was incubated at 22 °C for 2 h in dark and the fluorescence intensity was measured in a fluorescence spectrometer (FL-2000, Hitachi) at an emission and excitation wavelengths of 460 and 355 nm, respectively (n = 6).

As for catalase and superoxide dismutase (SOD) activity measurements, cells were collected by centrifugation at 2,000 rpm for 10 min at 4 °C. The cell pellet was lysed in cold 20 mM HEPES buffer (pH7.2). Then the solution was centrifuged at 10,000 rpm for 10 min at 4 °C. 10 μL the supernatant was mixed with 120 μL PBS (50 mM, pH 7.8), 30 μL of 130 mM methionine, 30 μL of 750 μM nitroblue tetrazolium (NBT), 30 μL of 100 μM EDTA-Na and 30 μL of 20 μM riboflavin. The reactive system was kept at 25 °C for 20 min under sunlight illumination. The OD value of the reactive system was finally measured at 560 nm for SOD assay (n = 6). Additionally, the catalase activity was detected using manufacturer’s protocol and the absorbance was monitored at 540 nm (n = 6).

### *In vivo* safety assessment

The median lethal dosage (LD50) of Z-ZnO/Cd(OH)Cl nanocomposites was determined on 6–8-weeks-old mice (BALB/c) without considering female or male, which were purchased from Shanghai SLAC Laboratory Animal Co., Ltd and fed autoclaved food and water ad libitum. All animal procedures were approved by the institutional animal care committee. All guidelines met the ethical standards required by law and also complied with the guidelines for the use of experimental animals in China. Z-ZnO/Cd(OH)Cl nanocomposites were ultrasonically dispersed into 1 mL normal saline and seven doses, including 5, 25, 50, 75, 100, 125 and 150 mg/kg body weight, were administrated i.v. to mice in seven groups (n = 10). The animals were carefully observed for any paradoxical reaction, toxic effect and distribution of death rate, immediately after dosing, during the subsequent 14 days. The median lethal dosage value was determined.

The different organs were carefully removed from each animal, fixed with 10% formalin, dehydrated in alcohol and then embedded in paraffin. After sectioning and staining with hematoxylin and eosin (H&E), the samples were examined to analyse the histological changes of the tissues.

Terminal Transferase dUTP Nick End Labeling (TUNEL) assay is a routine method used to detect DNA degradation in apoptotic cells and TUNEL kit was purchased from the Boehringer Mannheim GmbH. Brown is determined to be the positive apoptotic cells. 10 consecutive cells were observed and the number of positive cells in at least 1000 cells was counted, the apoptosis index (AI) was expressed as a percentage of the TUNEL-positive cells in the tumour cells.

### Pharmacokinetics and bio-distribution of nanocomposites *in vivo*

6–8-weeks-old mice (BALB/c) without considering female or male were administrated via the tail vein at the dose of 5 mg/kg (n = 5). At the given intervals (0, 3 min, 5 min, 10 min, 15 min, 30 min, 1 h, 6 h, 12 h, 1 d, 7 d, 14 d and 21 d), 0.5 mL blood sample was collected. Each blood sample was digested by 4.5 mL nitric acid, and the Zn and Cd levels were determined by AAS tests. The blank blood sample from mice was obtained and treated in the same way by injecting normal saline alone instead of nanocomposites. Pharmacokinetic analysis was performed on the plasma concentration-time profile.

At the same time, animals were sacrificed by cervical dislocation. The tissues of interest (including heart, liver, brain, spleen, kidney and lungs) were taken out, weighed and directly kept in liquid nitrogen. When Zn and Cd concentrations were analyzed, tissue was taken out and homogenized with an adequate amount of physiological saline. Then 5 mL nitric acid was added and the Zn and Cd levels were determined by AAS tests.

## Results and Discussion

Initial reports on the potential toxicity of nanomaterials promote the development of different modified nanomaterials as tools in the medicine field^16^,^17^,^42^,^43^,^44^. It is well known that Zn and Cd or their corresponding compounds can induce the cytotoxicity via different routes^38^,^39^,^40^,^41^. With our interest in the “drug combination” and “nanomedicine”, we envisaged that the incorporation of Cd(OH)Cl into ZnO would generate a new class of nanodrug with high-efficiency killing ability to cancer cells.

**Figure 1:**
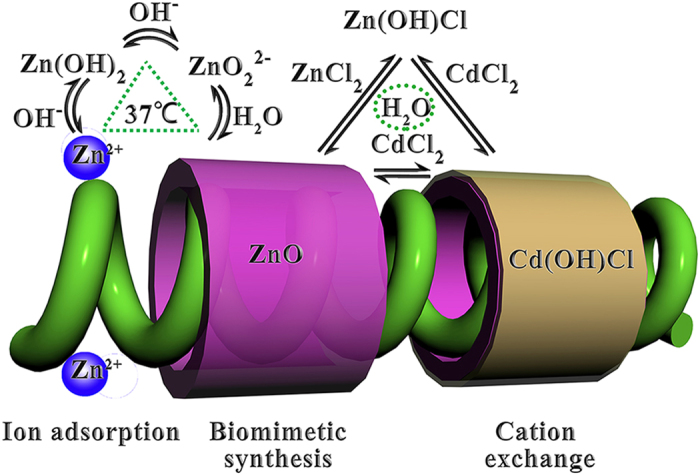
The green fabrication route of Z-ZnO/Cd(OH)Cl hierarchical nanocomposites.

**Figure 2:**
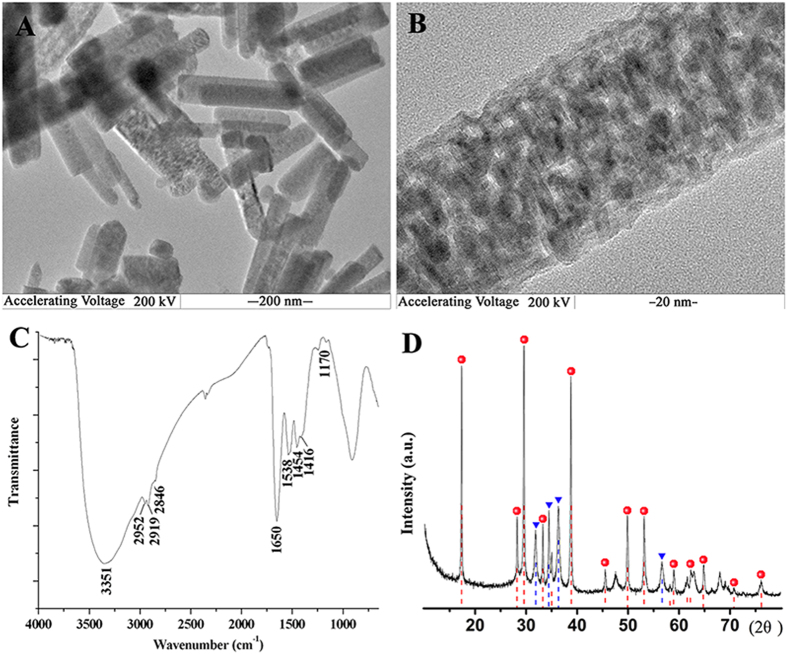
Morphological and structure analysis of typical Z-ZnO/Cd(OH)Cl hierarchical nanocomposites (12 h-ion exchange product). (**A**) TEM image; (**B**) magnification image; (**C**) FTIR spectrum; (**D**) XRD spectrum.

In this study, we designed the combination pathway of the biomimetic synthesis and ion-exchange technique to fabricate Z-ZnO/Cd(OH)Cl nanocomposites (Fig. 1)^45^,^46^. This route had two advantages. First, the whole synthesis route could be accomplished under the eco-friendly and gentle conditions, which would reduce the dubious factors from organic system and investment cost from special equipment. During the biomimetic synthesis process, the reactive system was 60% alcohol aqueous solution and zein was applied as the template to direct the synthesis of nanocomposites. The results revealed that zein could act as transport carrier in Ostwald ripening and induce the formation of rod-like nanocomposites^47^. By comparison with the control without zein, TEM images, FTIR spectra, XRD and TG/DTA curves clearly demonstrate the participation of zein in the ordered packaging of rod-like Z-ZnO/Cd(OH)Cl nanocrystals and the protein content gets to 10.25 ± 2.31% in nanocomposites (Fig. 2, Figures S1 and S2, see supporting information). Second, the ion exchange technique allowed to replace the first chemical with others, form a new composite with the initial morphology, and tune the ratio of different compositions by controlling the exchange time^48^. With the aid of XRD and AAS, we could easily monitor and quantify the ratio of ZnO and Cd(OH)Cl in nanocomposites (Figures S3 and S4, see supporting information).

By the biological analysis *in vitro*, both MTT determination and neutral red uptake assay indicate that Z-ZnO/Cd(OH)Cl nanocomposites can induce cytotoxicity to tumor cells as shown in Figure S5 and the inhibition rate can get to 87.2 ± 2.9% when only 5 ppm of nanocomposites are used. By the microscope techniques, we can find the nanocomposites at the surface and inner of tumor cells (Figure S6A,B). Generally, before nanomaterials get to targeting organs, nanomaterials must avoid to interacting with serum proteins and subsequent the capture from the reticuloendothelial system^49^,^50^,^51^. Meanwhile, vast literatures report that cells favor to rapidly absorb negatively charged nanoparticles and the affinity of nanoparticles to cellular membrane mainly turns on electrostatic interactions^51^,^52^,^53^,^54^. Based on this understanding, we determined the isoelectric points of nanocomposites in water with different pH values. The result demonstrates that rod-like Z-ZnO/Cd(OH)Cl nanocomposites show the electro-negativity when the environmental pH values vary from 5 to 9 (Figure S7 and see supporting information). In fact, the isoelectric points of native serum proteins vary from pH 4 to pH 7, and always show the negative charge in the physiological environment. Furthermore, the endothelial cells lining the inside of blood vessels also have an electro-negative surface^55^,^56^. In other word, the nanomaterials with a negative surface could have a relatively longer cycling period and a bigger chance to reach cancer cells than that with a positive surface. Once the non-specific attachment of nanoparticles to the cell membrane surface occurs via electrostatic interactions, it will cause a localized neutralization on the cell membrane and the reduced charge density may favor the adsorption of other free nanoparticles^52^. Besides, the adsorbed nanoparticles also tend to form a cluster due to their repulsive interactions with the large negatively charged domains on the cell surface^51^,^52^. The formed cluster further evokes a bending of the membrane favoring in turn pinocytosis, non-specific endocytosis or phagocytosis for cellular uptake^49^,^50^,^51^,^53^,^54^. For example, Wilhelm *et al.* studied the intracellular uptake of anionic superparamagnetic nanoparticles in different tumour cells and concluded that the high efficiency of anionic nanoparticles cell uptake could be attributed to the non-specific process and subsequently the formation of nanoparticles clusters^49^,^50^. After that, we checked the nucleic acids distribution of tumor cells after exposure to Z-ZnO/Cd(OH)C nanocomposites by specific staining technique with acridine orange. The results show that the uptaken nanocomposites can directly cause the disintegration of nuclear membranes and escape of nuclear acids (Figure S8 and see supporting information). At the same time, a real-time observation by SEM was performed after exposure tumor cells to nanocomposites for different periods (Figure S9A and see supporting information).Interestingly, we can capture the attachment of nanocomposites, formation of some smaller pores and cell membrane introcession on cell surface, and the disintegration of whole cell. The other evidence of the destructive effect of Z-ZnO/Cd(OH)Cl nanocomposites on cellular membrane system was from LDH release (Figure S9B). In order to verify the disintegration mechanism of cell membrane system, we analyzed the oxidative stress markers levels^57^. The higher level of MDA unambiguously shows that membrane oxidation occurres when cells are exposed to Z-ZnO/Cd(OH)Cl nanocomposites (Figure S10A). Additionally, it is noted the depletion of cell GSH, catalase and SOD, which are the main components of the cellular antioxidant system (Figure S10B–D).

**Figure 3:**
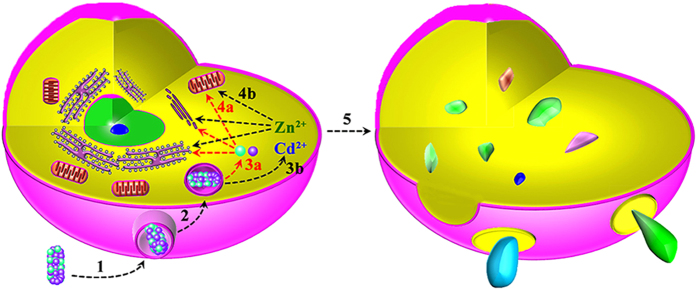
The possible pathways of lipid peroxidation-mediated disintegration of cells by Z-ZnO/Cd(OH)Cl hierarchical nanocomposites. (1) Attachment of nanocomposites to cell surface; (2) Uptake of nanocomposites; (3a and 3b) Release of metal ions or their corresponding chemicals; (4a and 4b) Targeting transportation of metal ions or their corresponding chemicals to cellular membrane system; (5) Destruction of cell membrane system.

Thereby, all these evidences clearly pointed to a conclusion that Z-ZnO/Cd(OH)Cl nanocomposites could kill cancer cells with high efficiency through oxidative stress mediated-membrane disintegration route. However, the detailed disintegration mechanism of the membrane system is unclear. Collective the abovementioned data, we deduced that the effect of Z-ZnO/Cd(OH)Cl nanocomposites on tumor cells might experience two pathways as shown in Fig. 3. Firstly, nanocomposites could act as Trojan horse-type carriers that enable the transport of ZnO and Cd(OH)Cl into cells (Step 1 and step 2, in Fig. 3)^58^. The untaken nanocomposites can release ZnO and Cd(OH)Cl with the enzymolysis of zein in endosomes^33^,^34^,^37^. Following that, the nanocomposites could directly destroy the membrane through lipid peroxidation (Via step 3a, step 4a, and to step 5, in Fig. 3)^57^. Secondly, it can’t be excluded that the released heavy metal ions intracellularly is also involved in the complex toxicity of nanocomposites^59^. According to the above-mentioned synthesis process in Fig. 1, the Z-ZnO/Cd(OH)Cl nanocomposites absolutely can release Zn^2+^ and Cd^2+^. Nowadays, an extensive amount of literatures have demonstrated that cadmium and zinc are toxic at low ionic concentrations^38^,^39^,^40^,^41^. For example, Shuilleabhain *et al.* found that zinc metal salts could arise the cytotoxicity in a zinc metal salt type- and cell type- dependent mode, with less than 2.5 mg/L of the maximum IC50 value^40^. Belyaeva *et al.* found that Cd^2+^ at micromolar concentrations induced both necrosis and apoptosis, inhibited cellular respiration and lowered ⊿ψ_mito_ in AS-30D hepatoma cells^41^. Therefore, it also might be reasonable that Z-ZnO/Cd(OH)Cl nanocomposites act as Trojan horse-type carriers for Zn^2+^ and Cd^2+^ during their anti-tumor process (Via step 3b, step 4b and to 5, in Fig. 3).

Observations on the high dispersion, stability and anti-tumor activity of Z-ZnO/Cd(OH)Cl hierarchical nanocomposites promoted us to focus on its behaviors *in vivo*, including the circulation half-life and tissue specificity. The acute toxicity evaluation of Z-ZnO/Cd(OH)Cl nano-hierarchical composites in mice indicated that no paradoxical reaction was observed after intravenous injection. Especially, no symptoms of toxicity, such as anorexia, severe diarrhea and weight loss, were evident at a dose of 5 mg/kg. The BALB/c mice appeared to be healthy throughout the experimental period. Moreover, H&E staining confirms that cell proliferation and division in all tissues have no obvious difference by comparison with the control group (Figure S11). At the same time, TUNEL assay reveals the same level at the apoptotic index of tissue cells in administration group and control group (Figure S12). However, the death of mice is observed when the dosage of nanocomposites is over 25 mg/kg (Table S1). The LD_50_ value of nano-composites administrated by i.v. is calculated to be 114.8 ± 3.6 mg/kg. After intravenous injection, the serum Zn concentration rapidly gets to the highest up to C_max_ 10.27 ± 1.22 μg/mL at 0.25 h and gradually decreases, and then recovers to the normal level at 1 h (Figure S13). The AUC_0–6_, T_1/2_ and MRT_0–6_ values are calculated to be 2.08 ± 0.25 μg h/mL, 0.36 ± 0.04 h and 0.59 ± 0.07 h, respectively, by non-compartmental analysis (Table 1). As for Cd, it has a relatively low serum concentration and quicker elimination rate, with 2.35 ± 0.19 μg/mL of C_max_ at 0.25 h, 0.17 ± 0.01 h of T_1/2_ and 0.25 ± 0.03 h of MRT_0–6_, respectively.

**Figure 4:**
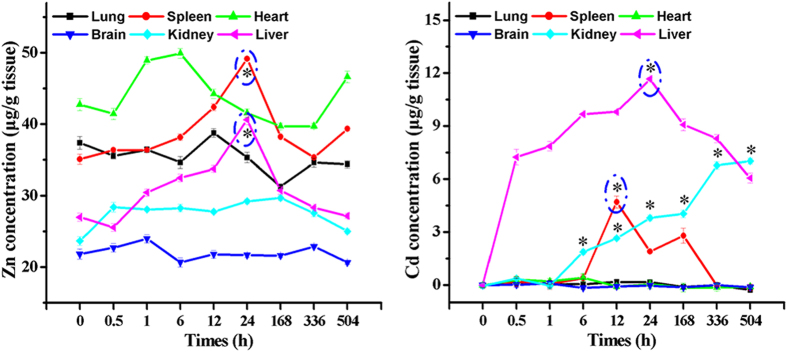
Dynamic distribution of Zn and Cd concentrations in different tissues (μg/g) after i.v. administration of Z-ZnO/Cd(OH)Cl hierarchical nanocomposites at a dose of 5 mg/kg (n = 5, *p* < 0.05).

Interestingly, after intravenous injection of Z-ZnO/Cd(OH)Cl nanocomposites, the significant increase of Zn and Cd contents only can be detected in the immune organs, such as liver, spleen and kidney (Fig. 4). Zn and Cd contents keep constant in other tissues and these results could indirectly prove the abovementioned effect of negative-charge surface of nanocomposites on its long cycling capacity. That is to say, when nanocomposites are delivered to the liver, spleen and kidney, they can be captured and phagocytized by the macrophage system^60^. This may be the main reason for the immune organs-targeting of Z-ZnO/Cd(OH)Cl nanocomposites. Summarily, the curative effect of the Z-ZnO/Cd(OH)Cl nanocomposites on the cancer of immune organs, such as liver cancer and renal carcinoma, is worth expecting. Moreover, it will be very interesting to find its targeting mechanism.

## Conclusions

Summarily, this paper proposed and verified the opinion about the introduction of “drug combination” into “nanomedicine”. Meanwhile, a green and gentle way was offered to synthesize the bioactive and bimetallic nanocomposites directly conjugated with biological species, with controllable metal proportions. Importantly, the synthesized Z-ZnO/Cd(OH)Cl nanocomposites showed the high anti-tumor activity at a low concentration (5 ppm), via lipid peroxidation-mediated disintegration of cellular membrane system. However, who on earth is the exact destroyer? nanocomposites or the released Zn^2+^ and Cd^2+^? It is still unclear. More interestingly, the Z-ZnO/Cd(OH)Cl nanocomposites could target to the immunity organs, which is worth expecting at the treatment of the corresponding cancers. However, the detailed targeting mechanism and curative effect are waiting to confirm. In addition, it is necessary to make a comprehensive evaluation on the safety of the resulting product, including immunotoxicity, genetic toxicity, reproductive and developmental toxicity, etc.

## Additional Information

**How to cite this article**: Wang, H.-J. *et al.* Green self-assembly of zein-conjugated ZnO/Cd(OH)Cl hierarchical nanocomposites with high cytotoxicity and immune organs targeting. *Sci. Rep.* **6**, 24387; doi: 10.1038/srep24387 (2016).

## Supplementary Material

Supplementary Information

## Figures and Tables

**Table 1:** Serum parameters of Z-ZnO/Cd(OH)Cl hierarchical nanocomposites in mice following intravenous injection at a dose of 5 mg/Kg using non-compartmental analysis with bolus IV administration (n = 5).

Elements	T_max_ (h)	C_max_ (μg/mL)	T_1/2_ (h)	MRT (h)	AUC_0–6h_ (μg h/mL)
Zn	0.25	10.27 ± 1.22	0.36 ± 0.04	0.59 ± 0.07	2.08 ± 0.25
Cd	0.25	2.35 ± 0.19	0.17 ± 0.01	0.25 ± 0.03	0.77 ± 0.08
