# Supplementary material for: Green self-assembly of zein-conjugated ZnO/Cd(OH)Cl hierarchical nanocomposites with high cytotoxicity and immune organs targeting
Source: Sci Rep. 2016 Apr 14;6:24387. doi: 10.1038/srep24387 (PMC4831000; doi:10.1038/srep24387)
Supplement: Supplementary Information [file srep24387-s1.doc]

Green self-assembly of zein-conjugated ZnO/Cd(OH)Cl hierarchical nanocomposites with high cytotoxicity and immune organs targeting

Hua-Jie Wang, ^[a, b]^ Ying Cao, *^[a]^ Cai-Feng Wang,^[a]^ Shi-Zhong Cui,^[a]^ Li-Wei Mi, *^[a]^ and Teruo Miyazawa, ^[a, b]^

^[a]^ Center for Advanced Materials Research, Zhongyuan University of Technology, No. 1 Huaihe Road, Xinzheng Shuanghu Economic Development Zone, Zhengzhou 451191, P. R. China.

^[b]^ New Industry Creation Hatchery Center (NICHe), Tohoku University, Sendai 980-0845, Japan

1. Results

In order to present the data clearly, zein-conjugated ZnO/Cd(OH)Cl hierarchical nanocomposites obtained by ion exchange for 0, 4, 8, 12, 24 and 48 h were abbreviated as Z-ZnO/Cd(OH)Cl^@0h^, Z-ZnO/Cd(OH)Cl^@4h^, Z-ZnO/Cd(OH)Cl^@8h^, Z-ZnO/Cd(OH)Cl^@12h^, Z-ZnO/Cd(OH)Cl^@24h^ and Z-ZnO/Cd(OH)Cl^@48h^, respectively.

*1.1. Design and green synthesis of Z-ZnO/Cd(OH)Cl hierarchical nanocomposites*

A combination route of the biomimetic method and ion-exchange technique was designed to fabricate Z-ZnO/Cd(OH)Cl hierarchical nanocomposites at gentle conditions. Firstly, the possible formation route of zein-conjugated ZnO hierarchical nano-structure could be illuminated based on the following three chemical equations and Scheme 1:

As described in the experimental section, Zn(OH)_2_ precipitate was formed quickly (*K*_sp_=10^-11^) (eq 1). However, Zn(OH)_2_ could be gradually converted into soluble ZnO_2_^2-^ by consuming hydroxyl anions in the reaction system (eq 2). At the same time, the resultant ZnO_2_^2-^ is instantaneously converted into ZnO solid phase (eq 3). Because the reaction system contains 60 % ethanol, it could deactivate the generating OH^-^ and ensure the reaction shifting to the right in eq 3.

Secondly, it has been well known about eq 4. In the present work, ZnCl_2_ was taken placed by CdCl_2_ and we immersed the obtained zein-conjugated ZnO nanorods into CdCl_2_ alcohol aqueous solution in order to obtained Z-ZnO/Cd(OH)Cl hierarchical nanocomposites. The possible formation route could be explained by eq 5 and eq 6. Briefly, zein-conjugated ZnO precipitation could transform into Zn(OH)Cl and Cd(OH)Cl in CdCl_2_ aqueous solution at pH 6.0 (eq 5). However, Zn(OH)Cl is very unstable in the presence of excess of CdCl_2_, which promote the ion exchange occurrence between Zn^2+^ and Cd ^2+^ (eq 6).

Figure S1 shows HR-TEM images of ZnO control without adding zein and Z-ZnO/Cd(OH)Cl^@0h^ composites. It can be seen that ZnO control without zein exhibits a ruleless and bulk structure, which is composed of nano-particles with about 4 nm in diameter (Figure S1A_1_ and S1A_2_). A further observation indicates that ZnO control has good crystalline and clear lattice fringes (Figure S1A_3_). The experimental lattice fringe spacing can be calculated to be 0.318 nm, which is in agreement with the standard *d* value for the (110) plane of ZnO. With the aid of zein, the obtained product has well dispersity and displays a regular and spindle-shape structure, with about 150 nm in diameter and 450 nm in length (Figure S1B_1_). A closer observation indicates that the spindle-shape structure is composed of nano-particles with about 5 nm in diameter (Figure S1B_2_). It is well known that zein has flattened cylinder structure with a length to width ration of ~2:1 and dimensions of ~6×3 nm. ^[10, 11]^ That is to say, zein acts as the structure-directing agent in this study. However, the lattice fringes indicate that it has a different growth mode, by comparison with the ZnO control. It has 0.259 nm of lattice fringe spacing and is corresponding to the (102) plane of ZnO (Figure S1B_3_). As for Z-ZnO/Cd(OH)Cl^@24h^ nanocomposites, they still have well dispersity but assemble into the rod-like structure, with about 72 nm in the diameter and 300 nm in the length (Figure 1A). Moreover, they also have the hierarchical structure and are composed of nano-particles with about 10 nm in diameter (Figure 1B).

The presence and content of zein in rod-like Z-ZnO/Cd(OH)Cl^@12h^ hierarchical nanocomposites were examined by FTIR spectra and TG/DSC analysis. Figure1C shows the FTIR spectra of Z-ZnO/Cd(OH)Cl^@12h^ nanocomposites. It can be seen that the peaks at 3351 cm^-1^, 2952 cm^-1^, 1650 cm^-1^ and 1538 cm^-1^ are clearly separated and can be assigned to the stretching vibration of hydroxyl group, amide A′, amide Ⅰ and amide Ⅱ, which are the typical protein absorption peaks. Other peaks observed at 2919 cm^-1^, 2846 cm^-1^ and 1454 cm^-1^ are correspond to C-H bonding due to the formation of co-ordination bond between zein and Zn^2+^ ions. Figure S2 shows the TG/DSC curves of rod-like Z-ZnO/Cd(OH)Cl^@12h^ hierarchical nanocomposites. The exothermic DSC peaks appear at 342 ℃ and 560 ℃, corresponding to the escape of several kinds of small molecules from the intramolecular disintegration of zein and combustion of residues. The total weight loss in TG curve gets to about 10.25 ± 2.31 % from 220 ℃ to 605 ℃ due to the thermal decomposition of zein.

*1.2. Monitoring on the synthesis proceeding of nano-hierarchical composites*

In order to verify the synthesis proceeding, the time-dependent evolution experiments, including XRD analysis and AAS test, were carried out. Figure S3 represents the XRD patterns of Z-ZnO/Cd(OH)Cl nanocomposites obtained by ion exchange for different time. It demonstrates that the obtained product is made of crystallized ZnO before ion exchange (0 h, Z-ZnO/Cd(OH)Cl^@0h^ nanocomposites). All characteristic peaks can be well assigned to the hexagonal-phase zincite reported in the literature (JCPDS card no. 36-1451). With the proceeding of ion exchange up to 4 h, the diffraction peak intensity of ZnO in Z-ZnO/Cd(OH)Cl^@4h^ is obviously decreased by comparison with that of Z-ZnO/Cd(OH)Cl^@0h^ nano-composites. At the same time, the diffraction peaks for Cd(OH)Cl also can be observed, suggesting the occurrence of ion exchange between Zn^2+^ and Cd^2+^. For example, the new peaks appear at 17.255°, 28.129°, 29.468°, 33.188°, 34.917°, 38.682°, 45.423°, 49.785°, 53.084°and 58.916°, which are corresponding to the crystal planes of 002, 100, 101, 102, 004, 103, 104, 110, 112 and 201 of hexagonal Cd(OH)Cl (JCPDS card 74-1047). However, the diffraction peaks of Zn(OH)Cl can’t be detected. It suggested that Zn(OH)Cl was only a transitory intermediate in the reactive system as expected and could be instantaneously transformed into Cd(OH)Cl through the cation exchange. With the further prolongation of ion-exchange time, the diffraction peak intensity of ZnO gradually weakens, but contrary to that of Cd(OH)Cl. The typical XRD pattern of Cd(OH)Cl appears after 24 h of ion exchange and almost no peaks for ZnO can be detected.

In order to quantify the ion-exchange process, AAS tests were carried out. As shown in Figure S4, the Zn and Cd contents in Z-ZnO/Cd(OH)Cl^@4h^ nanocomposites occupy 53.8 % and 21.9 %, respectively. 12 h later, the Zn content is reduced to 43.4 %. However, the Cd content reaches 27.8 %. With the further proceeding of ion exchange, Zn is gradually lost and only 10.4 % of Zn is remained in Z-ZnO/Cd(OH)Cl^@48h^ nanocomposites. Here, the change curve of Zn and Cd content clearly exhibited and quantified the process of ion exchange between Zn and Cd, which will be helpful to understand the synthesis mechanism.

*1.3. Cytotoxicity of Z-ZnO/Cd(OH)Cl hierarchical nanocomposites*

Figure S5 shows the killing ability of the precursor Z-ZnO nanocomposites (Z-ZnO/Cd(OH)Cl^@0h^), Z-ZnO/Cd(OH)Cl^@4h^, BPbS/Ag_2_S^@12h^ and BPbS/Ag_2_S^@24h^ nano-composites on PC12 cells on the basis of the MTT method and neutral red uptake assay. The MTT method was carried out to determine succinic dehydrogenase activity and reflected the function of living-cell’s mitochondria. At the same time, the neutral red uptake assay was applied to determine the accumulation of neutral red dye in the lysosomes of viable, uninjured cells. As shown in Figure S5, Z-ZnO/Cd(OH)Cl nanocomposites have a high killing capacity on tumor cells. Both the mitochondrial activity and the uptaken neutral red in lyosomes decrease with the increase of Z-ZnO/Cd(OH)Cl nanocomposites. Z-ZnO/Cd(OH)Cl^@0h^ nanocomposites show the significantly weaker cytotoxicity than the ion-exchange products. Among the Z-ZnO/Cd(OH)Cl nanocomposites, no significant difference was observed. All of them exhibits high killing activity and the inhibition rate still can get to 87.2±2.9 % % when only 5 ppm of nanocomposites are used.

*1.4. Destructive evidences of cell membrane system*

Figure S6 shows the destructive evidences of Z-ZnO/Cd (OH)Cl nanocomposites on cellular membrane systems. The membrane surface structures were magnified by SEM. As shown in Fig. S6A, the cells form nearly monolayer in the absence of any nanocomposites, develop characteristic pseudopodia and cell membrane keep integrate (Figure S6A_1_). However, the phenomenon changes greatly after exposure to nanocomposites. The observed cells lose their typically morphological characteristic and contract to some extent. The cell membranes are destroyed completely and cells lose their content and become compressed. Besides, lots of holes are formed on the cells surface (Figure S6A_2_-S6A_4_).

Figure S6B further exhibits the inner structure of cells after exposure to Z-ZnO/Cd(OH)Cl nanocomposites by TEM observation. The black dots clearly appear in cells and near nuclear membranes. Cell membrane loses its integrity and lots of cytoplasm escape. Moreover, small pores also can be observed on nuclear membrane.

As shown in Figure S7, the zeta potential of Z-ZnO/Cd(OH)Cl nanocomposites varies from -18.9 to -29.9 when the pH values range from 5.0 to 9.0. The ion-exchange time has no significant effect on the surface charge of the obtained Z-ZnO/Cd(OH)Cl nanocomposites.

Based on the special staining action of acridine orange on nucleic acids, it can be seen that the cells grow well in the absence of any nanomaterials and the cell number is the most compared with that in other groups (Figure S8A_1_). Besides, cells develop a spindle-like morphology and produce long neuritis. A clear color contrast between green nucleus structure and yellow cytoplasm can be observed in each cell. After exposure to Z-ZnO/Cd(OH)Cl nanocomposites, the cell number significantly decreases but no significant difference among three groups could be detected (Figure S8A_2_-S8A_4_). As for Z-ZnO/Cd(OH)Cl^@4h^ exposure group, many cells have a black nuclear region. There are still a few cells that have an olivine nuclear and yellow cytoplasm structure (Figure S8A_2_). However, all cells loss their nuclear after exposure to Z-ZnO/Cd(OH)Cl^@12h^ for 48 h (Figure S8A_3_). As for Z-ZnO/Cd(OH)Cl^@24h^ treatment group, the situation is the same as that in Z-ZnO/Cd(OH)Cl^@4h^ treatment group (Figure S8A_4_).

A real-time observation by SEM was performed after exposure tumor cells to nanocomposites for different periods (Figure S9A).Interestingly, we can capture the attachment of nanocomposites, formation of some smaller pores and cell membrane introcession on cell surface, and the disintegration of whole cell. The other evidence of the destructive effect of Z-ZnO/Cd(OH)Cl nanocomposites on cellular membrane system was from LDH release (Figure S9B). It can be seen that Z-ZnO/Cd(OH)Cl^@0h^ only shows a slight effect on the LDH leakage when its concentration gets to 100 ppm. As for Z-ZnO/Cd(OH)Cl nanocomposites, a significant LDH leakage was observed.

*1.5. Oxidative stress markers*

The first oxidative stress marker was MDA concentration, which was an oxidized product of poly-unsaturated fatty acids (Figure S10A). A significant increase in MDA formation can be observed after exposure to 5 ppm of Z-ZnO/Cd(OH)Cl nano-composites (*p*<0.05). Moreover, the relative content of the released MDA gets to 11.7, 10.9 and 21.6 times higher in Z-ZnO/Cd(OH)Cl^@4h^, BPbS/Ag_2_S^@12h^ and BPbS/Ag_2_S^@24h^ group, respectively, than that in the control group. Besides, other oxidative stress markers were from anti-oxidation protection system, including GSH, catalase and SOD (Figure S10B-S10D). We can find that cellular GSH, catalase and SOD levels are significantly depleted.

*1.6. Acute toxicity and in vivo security*

The understanding on the pharmacokinetics and bio-distribution of nano-drug is currently a focus of attention, linked to the development of their clinical application. Observations on the high dispersion, stability and antineoplastic activity of Z-ZnO/Cd(OH)Cl nanocomposites promoted us to focus on its behaviors *in vivo*, including the circulation half-life and tissue specificity. Firstly, the acute toxicity evaluation of Z-ZnO/Cd(OH)Cl nanocomposites *in vivo* was carried out in mice. In the first day after i.v. administration of Z-ZnO/Cd(OH)Cl nano-hierarchical composites, no paradoxical reaction was observed in all groups. Especially, no symptoms of toxicity, such as anorexia, severe diarrhea and weight loss, are evident at 5 mg/kg, and the BALB/c mice appear to be healthy throughout the experimental period. Moreover, H&E staining confirms that cell proliferation and division in all tissues have no obvious difference by comparison with the control group (Figure S11). At the same time, TUNEL assay reveals the same levels at the apoptotic index of tissue cells in administration group and control group (Figure S12). However, the death of mice was observed from the second day when the dosage was over 25 mg/kg, but the death rate differed from the administered dosage as shown in Table S1. The LD_50_ value of nano-composites administrated by i.v. was calculated to be 114.8±3.6 mg/kg.

| **Table S1.** The death number and time of BALB/c mice after i.v. administration of Z-ZnO/Cd(OH)Cl hierarchical nanocomposites at different dosages (n=10). The test was repeated three times | | | | | | | |
| --- | --- | --- | --- | --- | --- | --- | --- |
| Dose (mg/kg) | 5 | 25 | 50 | 75 | 100 | 125 | 150 |
| Death number | 0 | 1 | 1 | 2 | 2 | 5 | 8 |
| Death time (h) | - | 30 | 36 | 96 | 68 | 162-168 | 158 |
|  | | | | | | | |


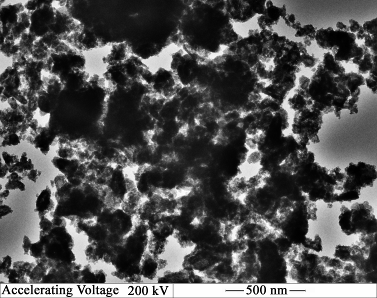

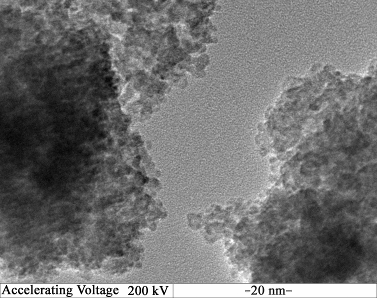

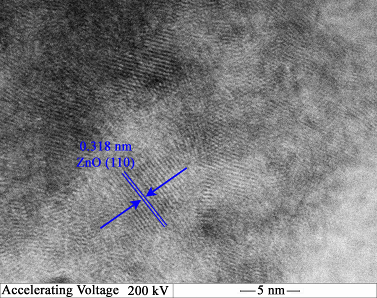

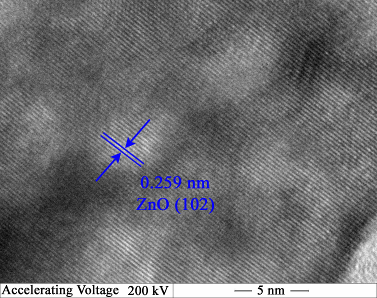

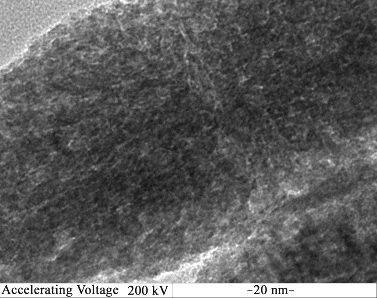


**B_3_**


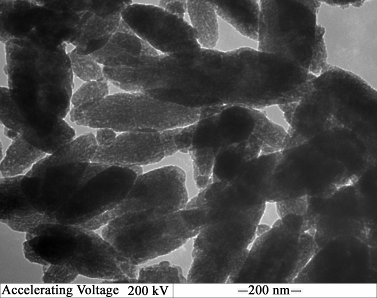


**A_1_**

**A_2_**

**B_1_**

**B_2_**

**A_3_**

**Figure S1**. HR-TEM images of ZnO control without adding zein (A) and zein-conjugated ZnO hierarchical nanospindles (Z-ZnO/Cd(OH)Cl^@0^ hierarchical nanocomposites) (B).


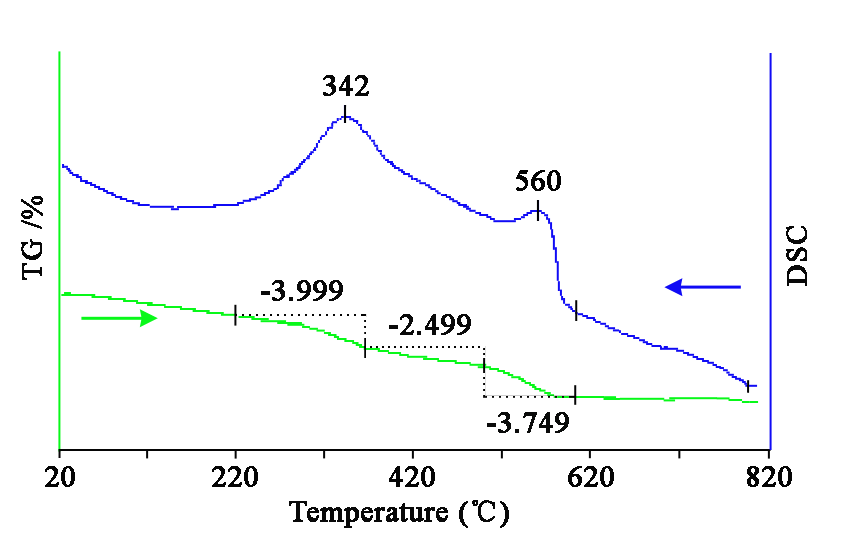


**F****igure S2**. TG/DSC curve of Z-ZnO/Cd(OH)Cl^@12^ hierarchical nanocomposites.


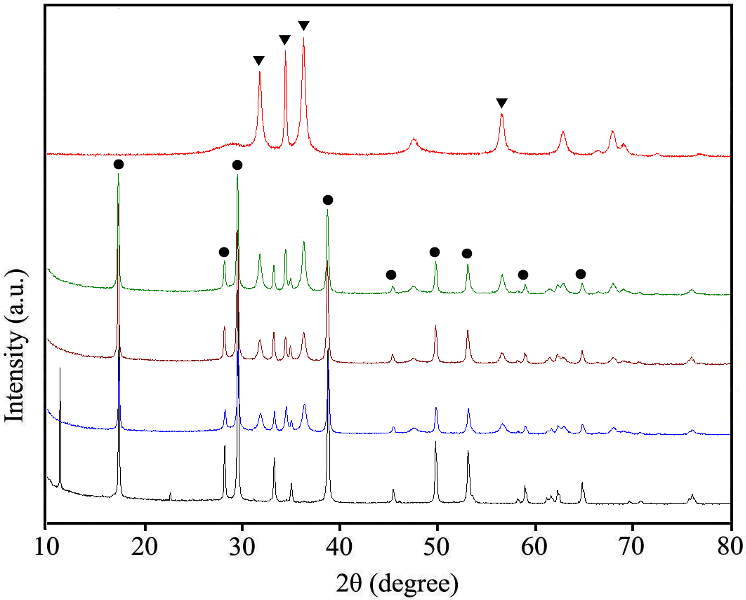


**4h**

**8h**

**12h**

**24h**

**0h**

**Figure S3**. XRD patterns of Z-ZnO/Cd(OH)Cl hierarchical nanocomposites obtained by a combination route of the biomimetic method and ion-exchange technique with different ion-exchange time.

**
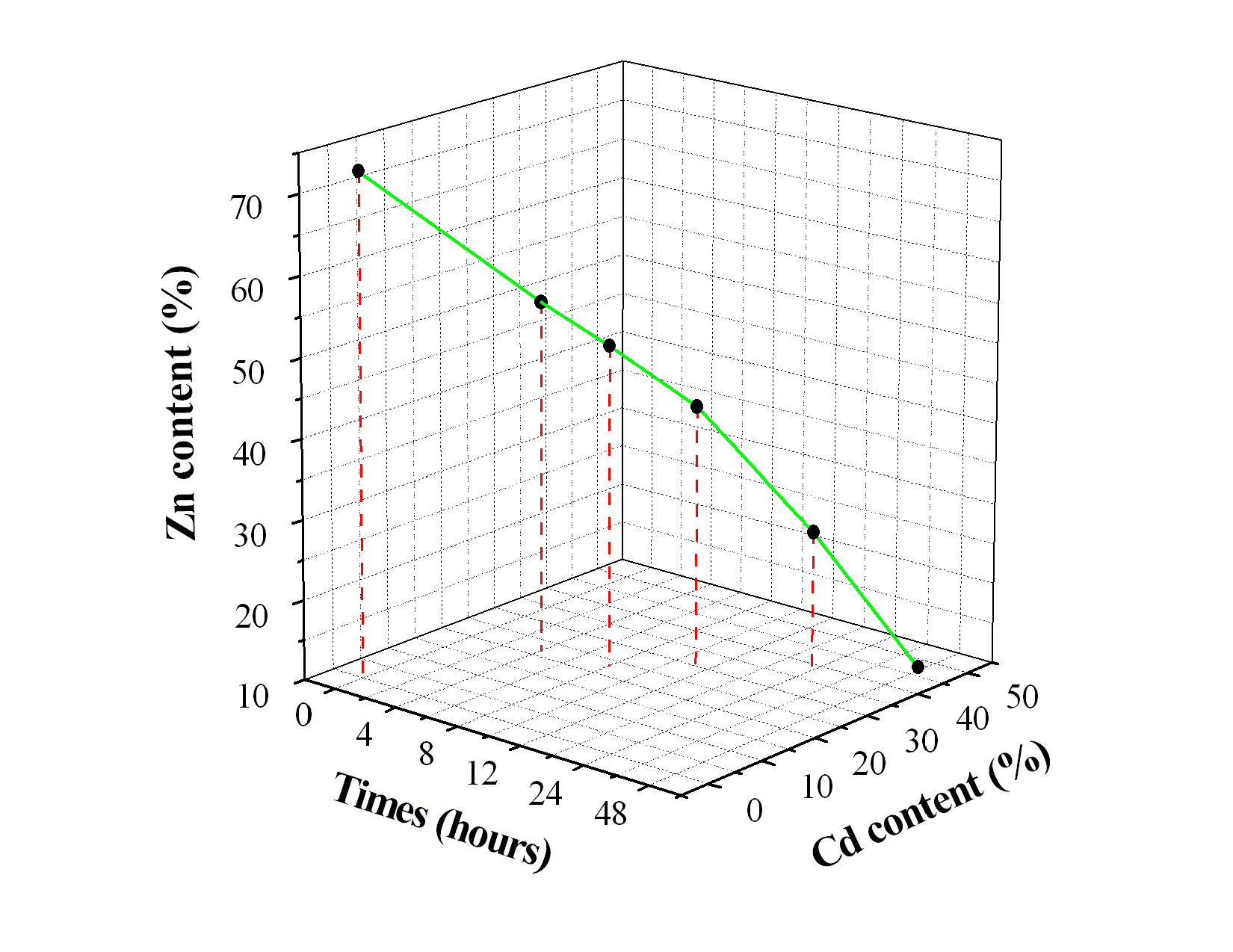
**

**Figure S4**. Atomic absorption spectroscopy analysis on the change of Zn and Cd contents in Z-ZnO/Cd(OH)Cl hierarchical nanocomposites obtained by a combination route of the biomimetic method and ion-exchange technique with different ion-exchange time.


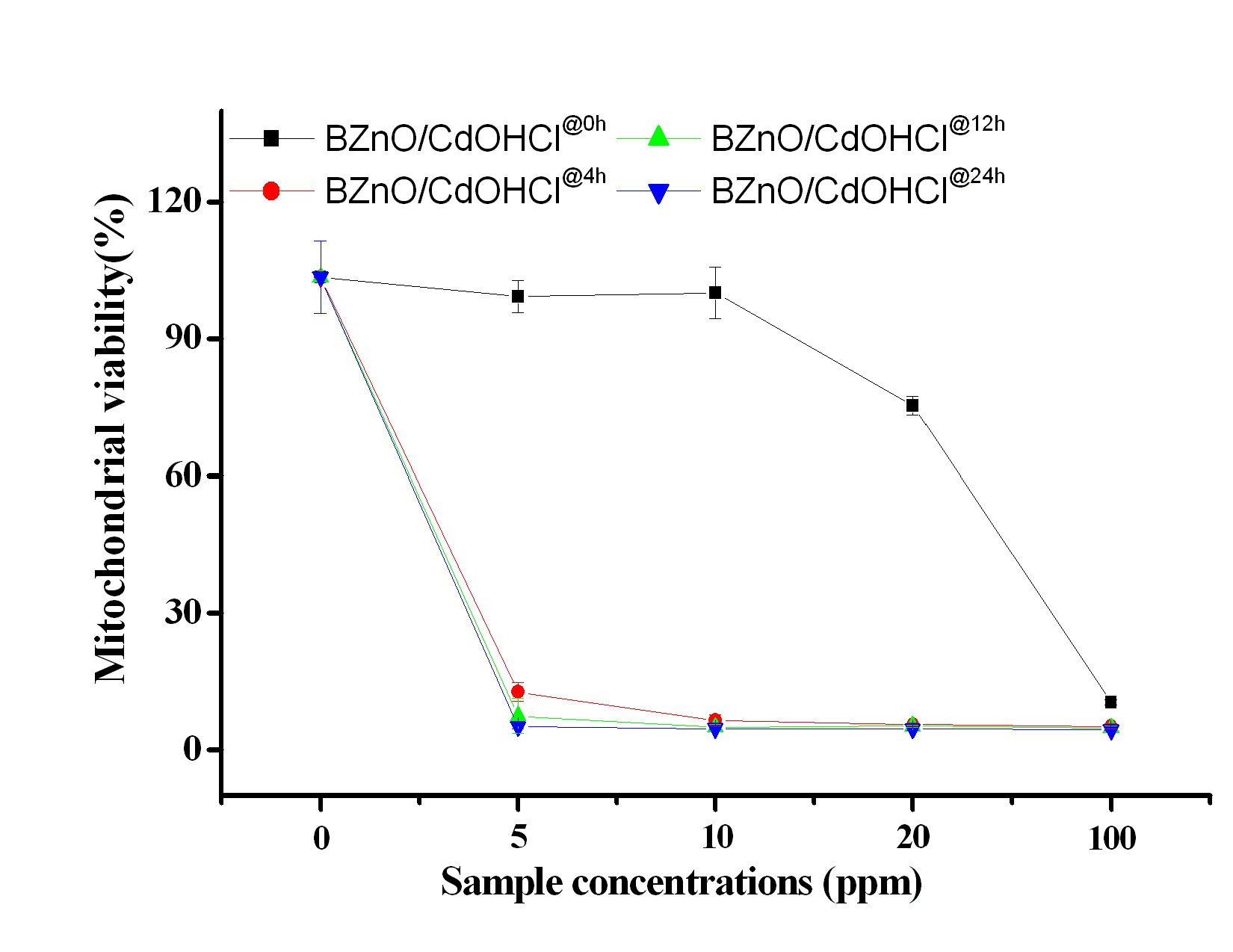

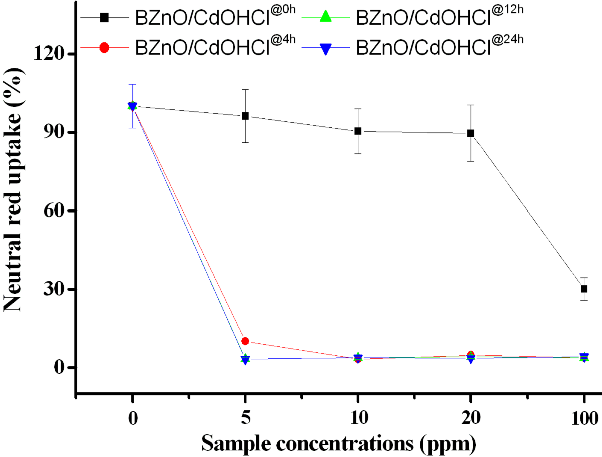


**Figure S5**. Cytotoxicity analysis of Z-ZnO/Cd(OH)Cl hierarchical nanocomposites on PC12 cells. (A) Mitochondrial viability measurement by MTT method; (B) Neutral red uptake assay.


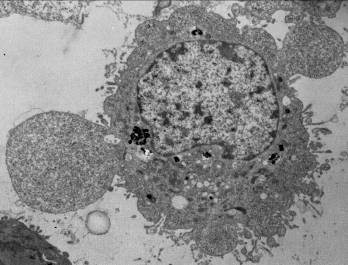

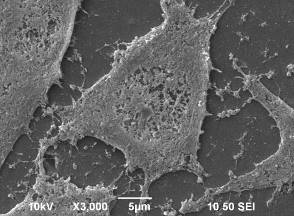

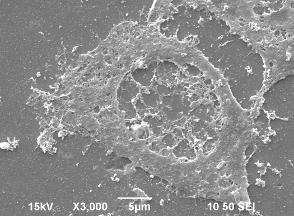

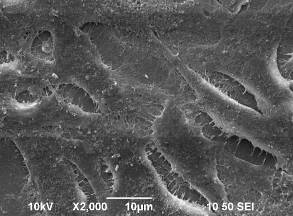

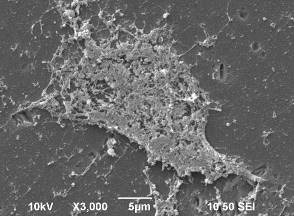


**A_1_**

**A_2_**

**A_3_**

**A_4_**


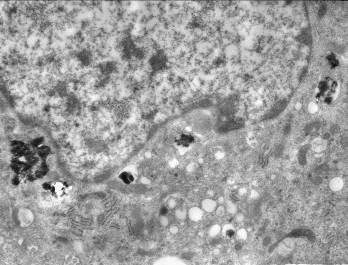


**B**

**Figure S6**. Destructive evidences of Z-ZnO/Cd (OH)Cl nanocomposites on cellular membrane systems. (A) SEM observation for outer detection of cell surface; (B) Cellular TEM image for localization of ZnO/Cd(OH)Cl^@12h^ nanocomposites and detection of membrane integrity; (1) Normal group; (2) ZnO/Cd(OH)Cl^@4h^; (3) ZnO/Cd(OH)Cl^@12h^ and (4) ZnO/Cd(OH)Cl^@24h^ hierarchical nanocomposites.

**Figure S7**. Influence of pH values on zeta potential of Z-ZnO/Cd(OH)Cl^@4h^ (◆), Z-ZnO/Cd(OH)Cl^@12h^ (△) and Z-ZnO/Cd(OH)Cl^@24h^ (○) hierarchical nanocomposites.


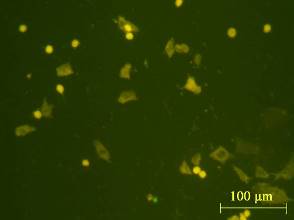

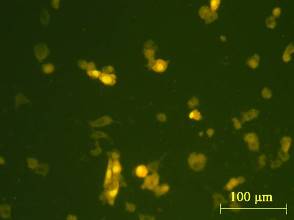

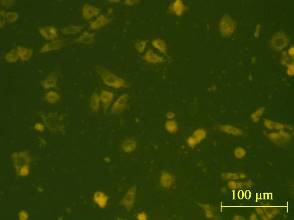

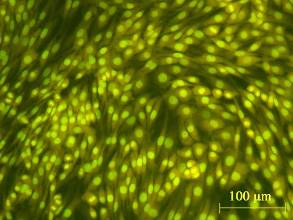


**A**

**B**

**C**

**D**

**Figure S8**. Fluorescence microscope observation of nucleic acids distribution after specific staining with acridine orange. (1) Normal group; (2) ZnO/Cd(OH)Cl^@4h^; (3) ZnO/Cd(OH)Cl^@12h^ and (4) ZnO/Cd(OH)Cl^@24h^ hierarchical nanocomposites.


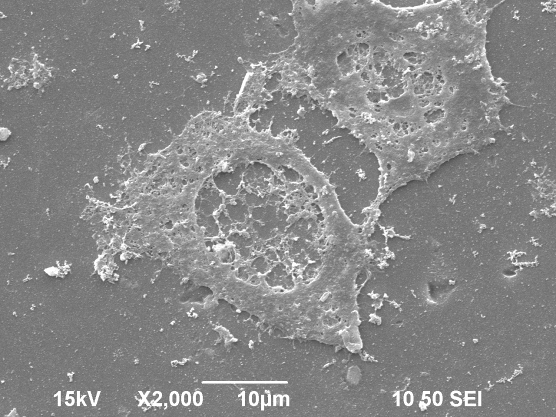

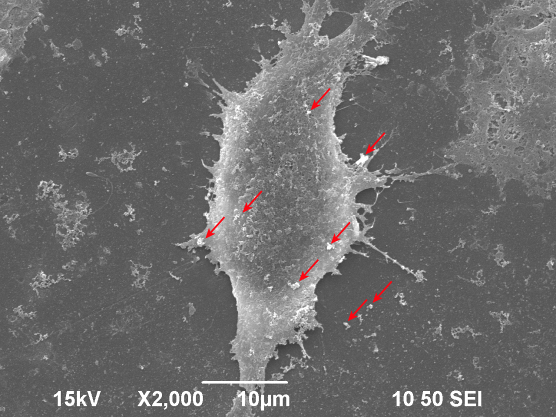

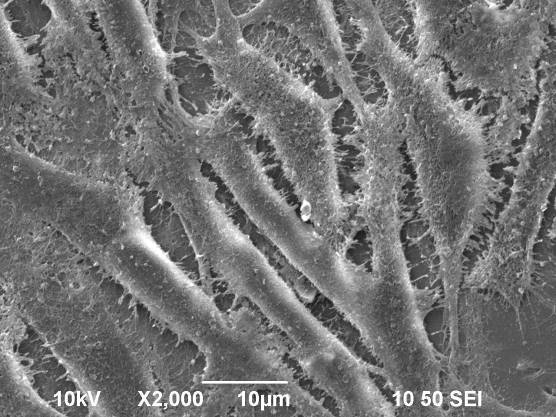

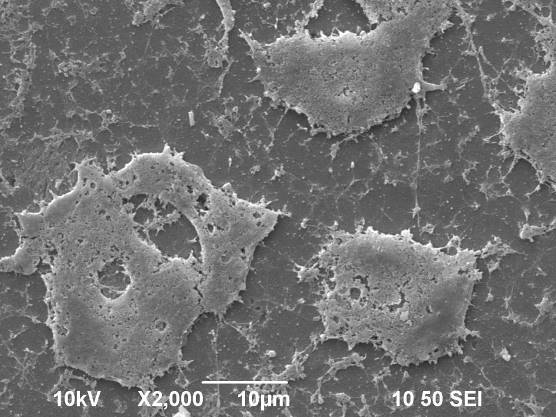

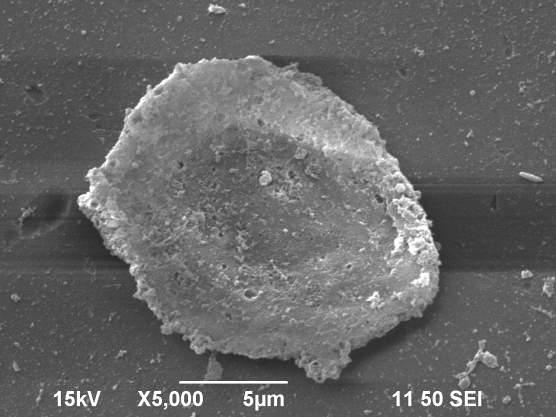


**A_1_**

**A_2_**

**A_3_**

**A_4_**

**A_5_**

**B**


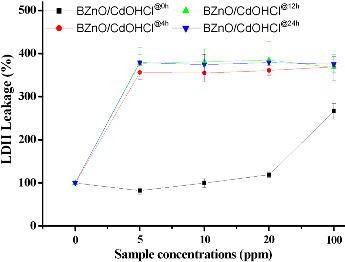


**Figure S9.** The death process of PC 12 cells (A) and LDH release (B) under the effect of Z-ZnO/Cd(OH)Cl hierarchical nanocomposites. (1) Normal cells; (2) The attachment of nanocomposites on cells; (3) The formation of introcession on the surface of cells; (4) The formation of smaller pores on the surface of cells; (5) The disintegration of cells.


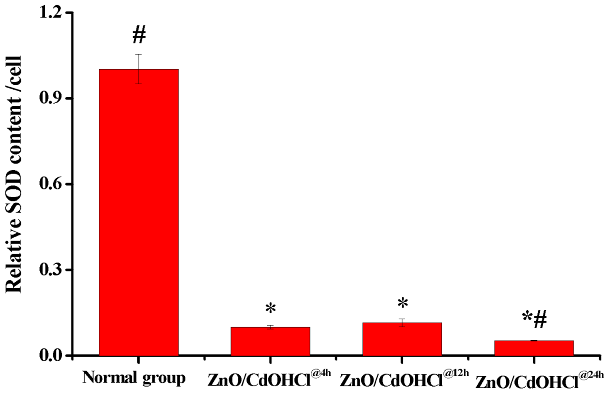

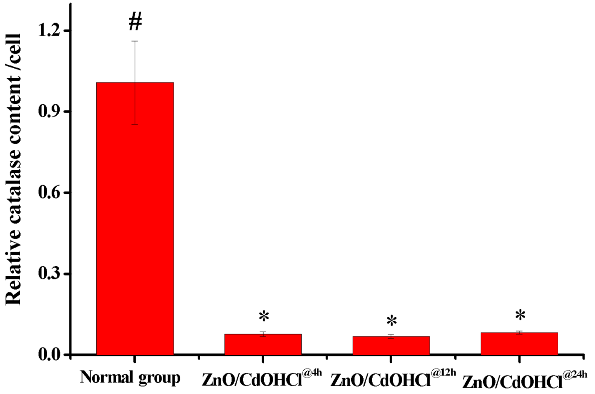

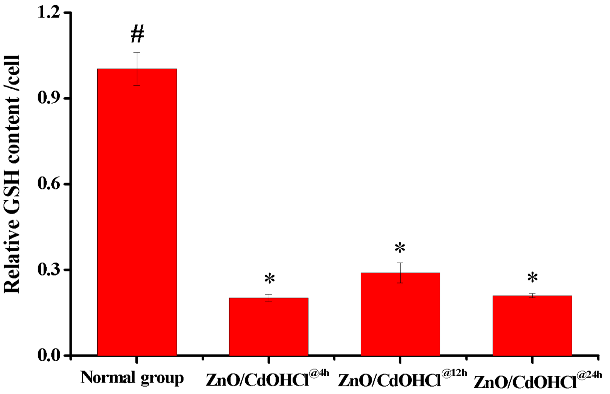

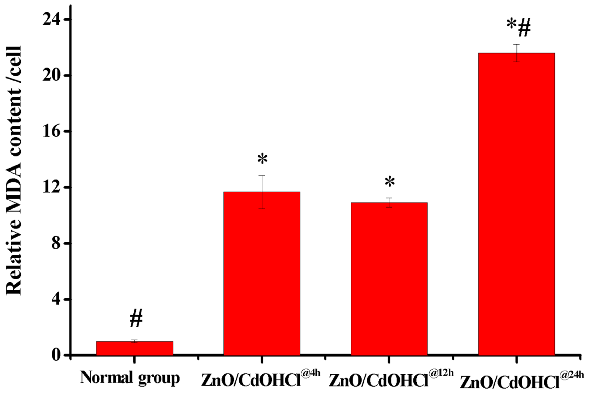


**A**

**B**

**D**

**C**

**Figure S10**. Cellular responses to 5 ppm of Z-ZnO/Cd(OH)Cl hierarchical nanocomposites on the basis of oxidative stress markers. The Y axis represents the relative MDA (A), GSH (B), Catalase (C) and SOD (D) contents of each cell. The value of normal group was set as 1. “*” and “#” mean the significant difference compared with normal group or other groups.


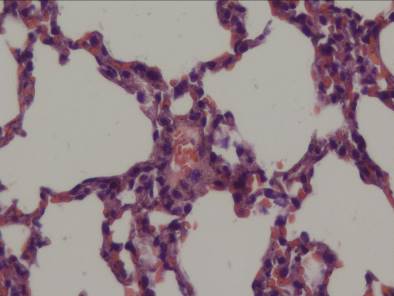

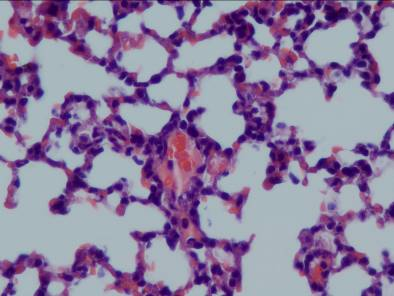

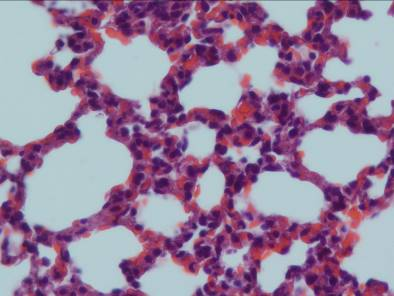

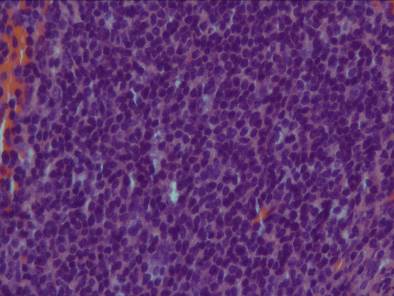

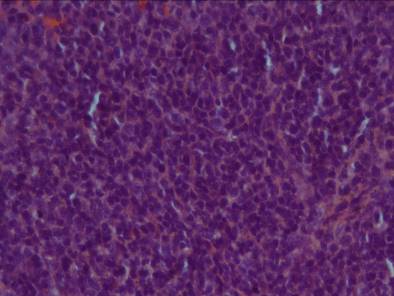

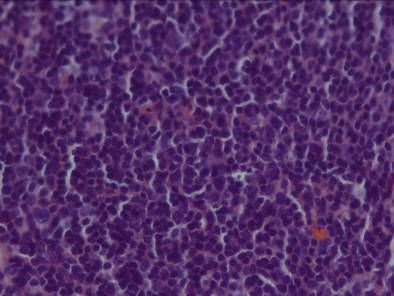

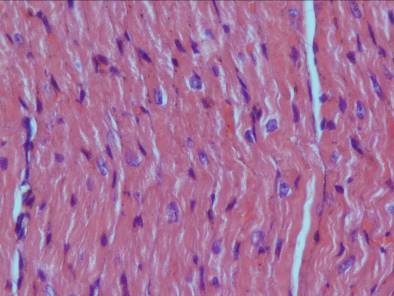

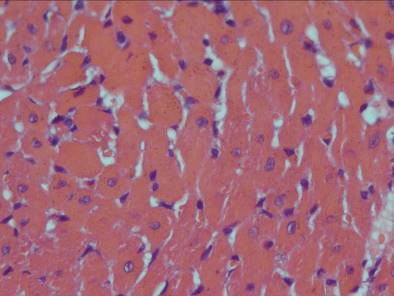

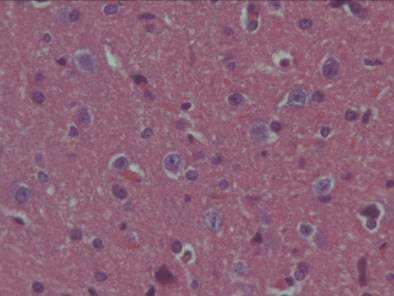

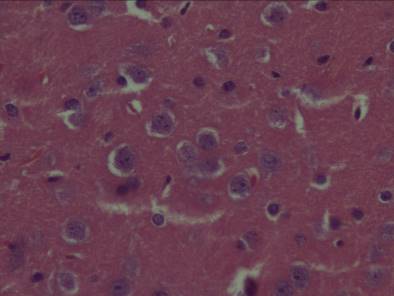

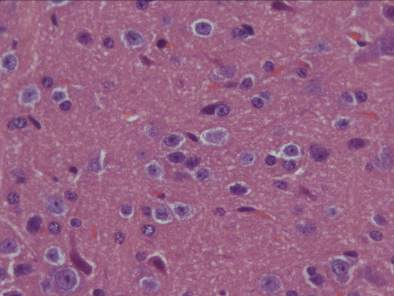

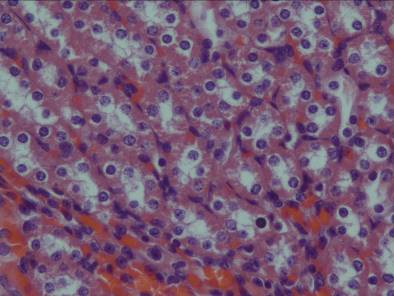

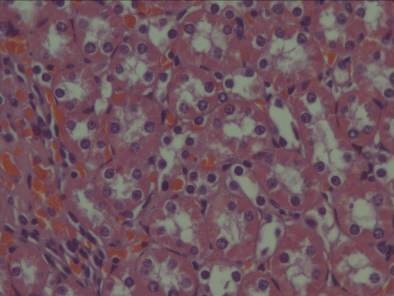

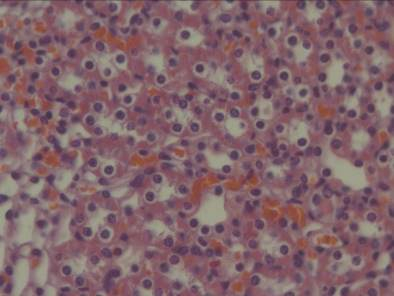

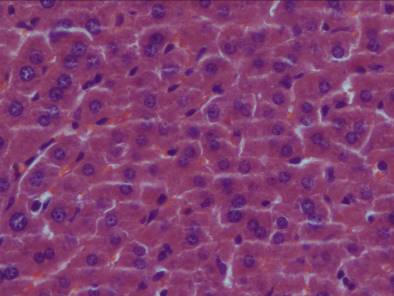

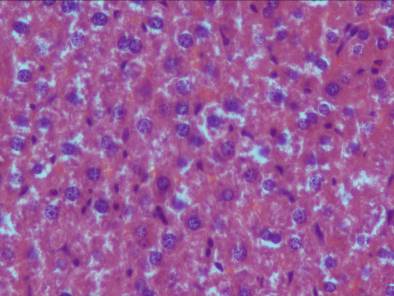


**Lung**

**kidney**

**Brain**

**Spleen**

**Heart**

**Liver**


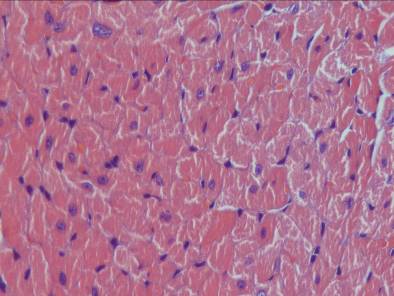

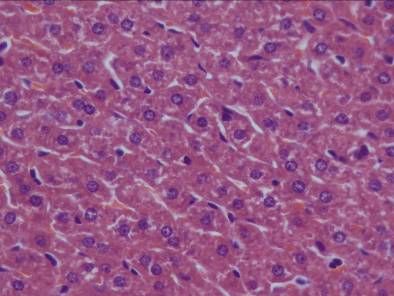


**C_1_**

**B_1_**

**A_1_**

**A_2_**

**A_3_**

**B_2_**

**B_3_**

**C_2_**

**C_3_**

**D_1_**

**D_2_**

**D_3_**

**E_1_**

**E_2_**

**F_1_**

**F_2_**

**E_3_**

**F_3_**

**Figure S11**. H&E staining on different tissues of BALB/c mice after i.v. administration of Z-ZnO/Cd(OH)Cl nanocomposites for 0 (1), 24 hours (2) and 1week (3) at a dose of 5 mg/kg.


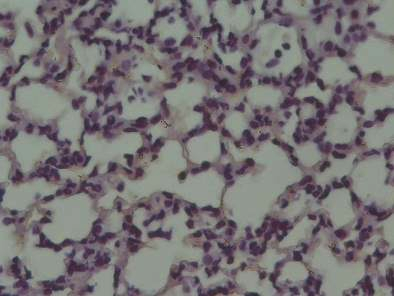

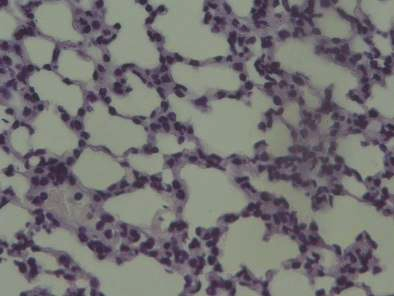

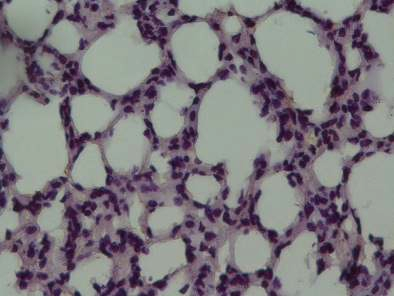

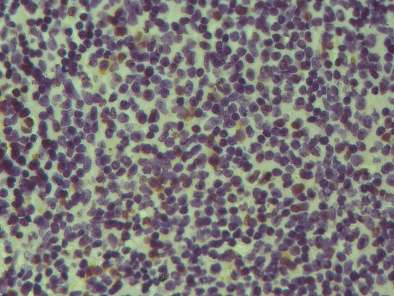

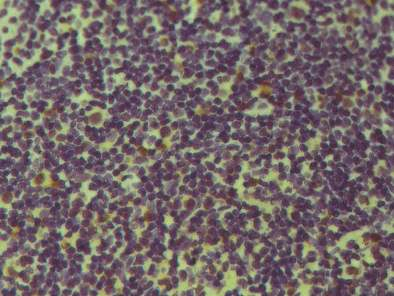

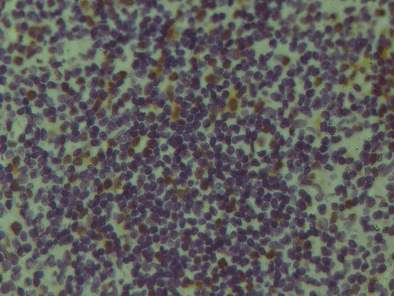

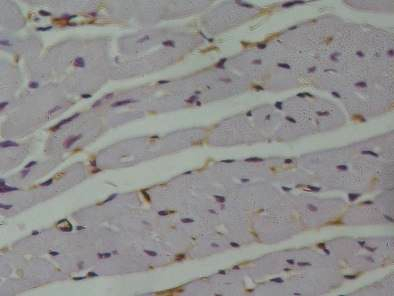

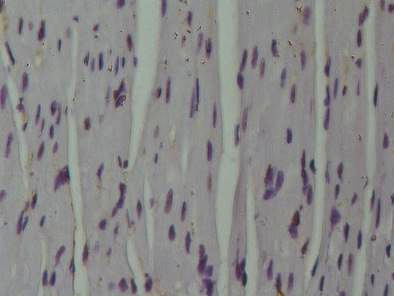

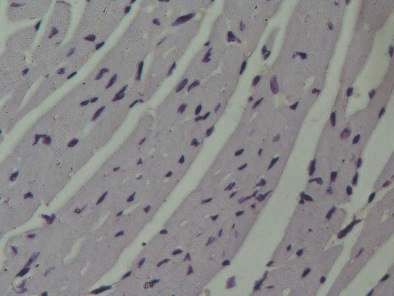

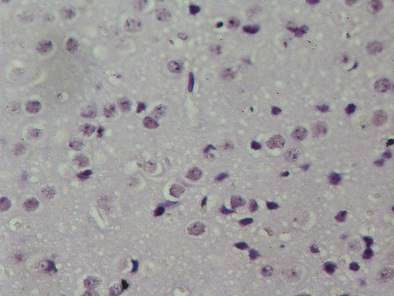

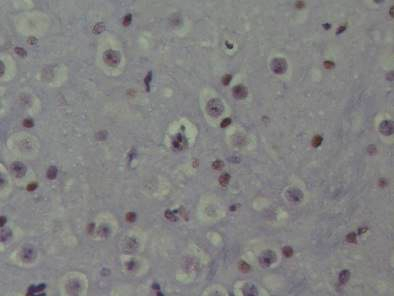

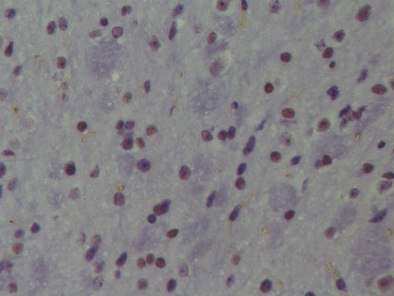

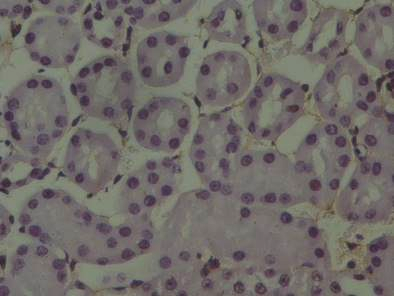

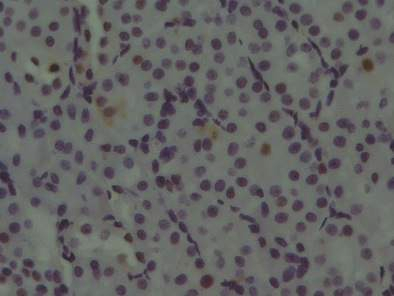

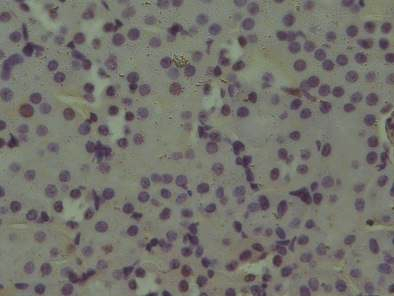

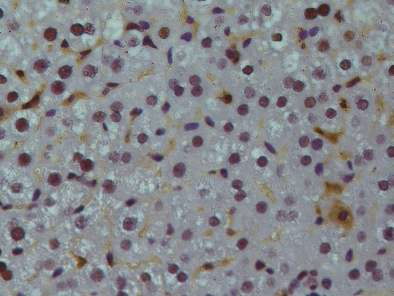

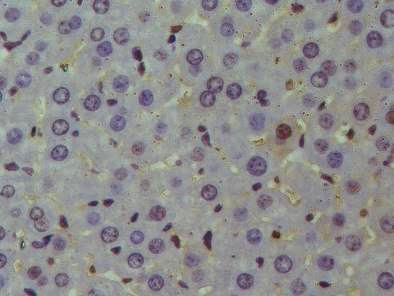

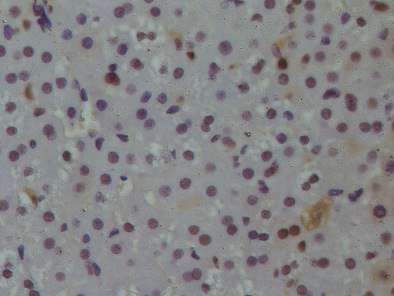


**kidney**

**A_3_**

**B_3_**

**C_3_**

**E_3_**

**F_3_**

**D_3_**

**D_2_**

**E_2_**

**F_2_**

**D_1_**

**E_1_**

**F_1_**

**Lung**

**Heart**

**Brain**

**Spleen**

**Liver**

**A_2_**

**B_2_**

**C_2_**

**C_1_**

**B_1_**

**A_1_**

**Figure S12**. TUNEL assay on different tissues of BALB/c mice after i.v. administration of Z-ZnO/Cd(OH)Cl nanocomposites for 0 (1), 24 hours (2) and 1week (3) at a dose of 5 mg/kg.


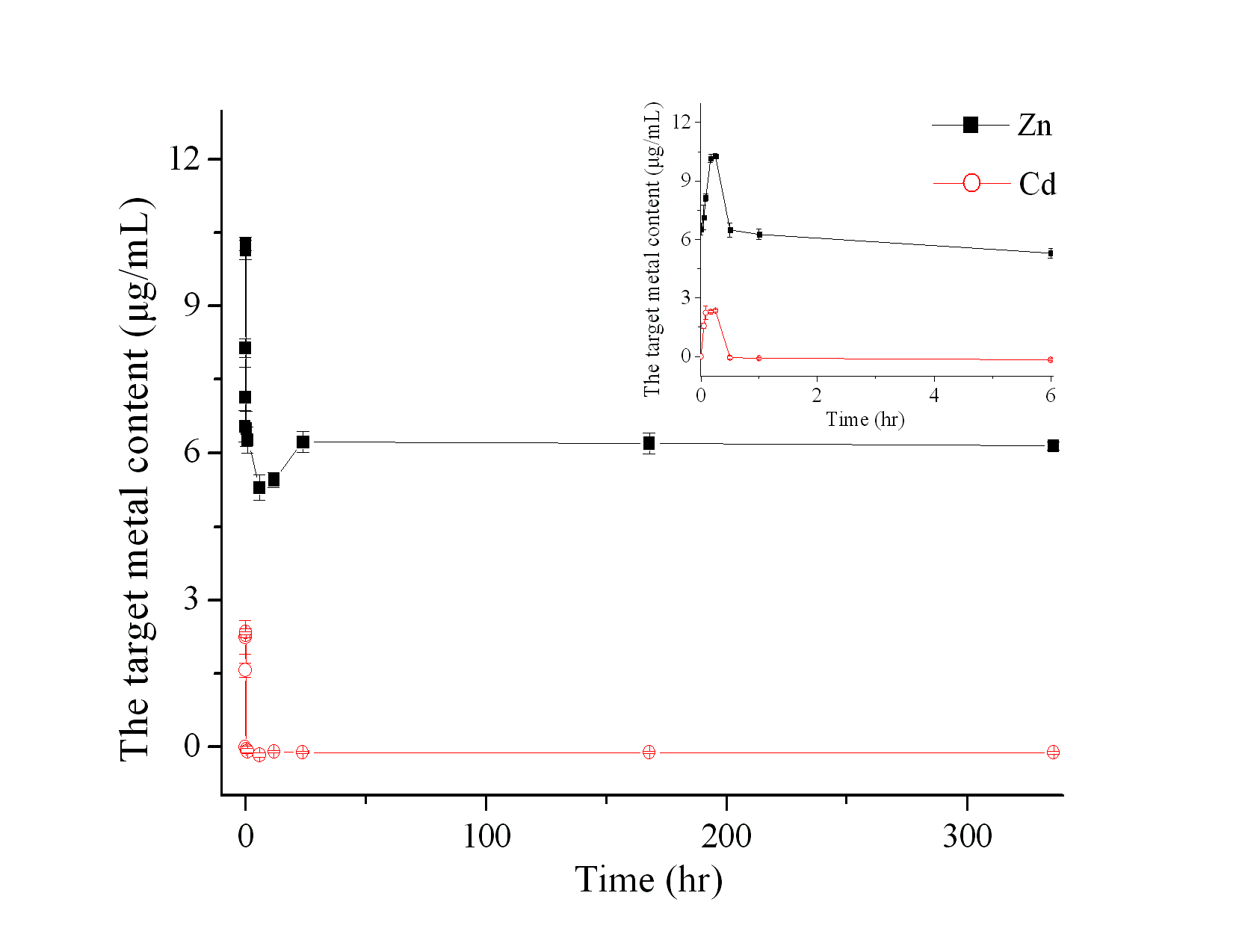


**Figure S13.** The serum concentration-time profiles of Zn and Cd after intravenous injection of Z-ZnO/Cd(OH)Cl nanocomposites at a dose of 5 mg/kg.
